# Interpretation of CVD risk predictions in clinical practice: Mission impossible?

**DOI:** 10.1371/journal.pone.0209314

**Published:** 2019-01-09

**Authors:** G. R. Lagerweij, K. G. M. Moons, G. A. de Wit, H. Koffijberg

**Affiliations:** 1 Julius Center for Health Sciences and Primary Care, University Medical Center, Utrecht, the Netherlands; 2 Dutch Heart Institute, Utrecht, the Netherlands; 3 Centre for Nutrition, Prevention and Healthcare, National Institute for Public Health and the Environment, Bilthoven, the Netherlands; 4 Department of Health Technology & Services Research, MIRA Institute for Biomedical Technology and Technical Medicine, University of Twente, Enschede, The Netherlands; University of Sydney, AUSTRALIA

## Abstract

**Background:**

Cardiovascular disease (CVD) risk prediction models are often used to identify individuals at high risk of CVD events. Providing preventive treatment to these individuals may then reduce the CVD burden at population level. However, different prediction models may predict different (sets of) CVD outcomes which may lead to variation in selection of high risk individuals. Here, it is investigated if the use of different prediction models may actually lead to different treatment recommendations in clinical practice.

**Method:**

The exact definition of and the event types included in the predicted outcomes of four widely used CVD risk prediction models (ATP-III, Framingham (FRS), Pooled Cohort Equations (PCE) and SCORE) was determined according to ICD-10 codes. The models were applied to a Dutch population cohort (n = 18,137) to predict the 10-year CVD risks. Finally, treatment recommendations, based on predicted risks and the treatment threshold associated with each model, were investigated and compared across models.

**Results:**

Due to the different definitions of predicted outcomes, the predicted risks varied widely, with an average 10-year CVD risk of 1.2% (ATP), 5.2% (FRS), 1.9% (PCE), and 0.7% (SCORE). Given the variation in predicted risks and recommended treatment thresholds, preventive drugs would be prescribed for 0.2%, 14.9%, 4.4%, and 2.0% of all individuals when using ATP, FRS, PCE and SCORE, respectively.

**Conclusion:**

Widely used CVD prediction models vary substantially regarding their outcomes and associated absolute risk estimates. Consequently, absolute predicted 10-year risks from different prediction models cannot be compared directly. Furthermore, treatment decisions often depend on which prediction model is applied and its recommended risk threshold, introducing unwanted practice variation into risk-based preventive strategies for CVD.

## Introduction

Reduction of cardiovascular disease (CVD) burden, i.e. at population level, is commonly accomplished using preventive strategies (like lifestyle and dietary advice or preemptive drug treatment) in individuals with marked elevations in risk factors, e.g. low-density lipoprotein (LDL), or a high predicted CVD risk based on a combination of risk factors [1]. Identification of high risk individuals is often achieved using CVD risk prediction models of which over 360 different variants have been published as of 2016 [2]. However, different models may predict multiple and often different CVD outcomes or sets of outcomes (as is the case in model with composite endpoints) [2–4]. These differences in predicted outcomes may result in large variation in CVD risk estimates. Consequently, it is unclear to what extent the predicted CVD risks obtained from different prediction models are comparable and can be interpreted similarly in clinical practice [4–7].

The large variation in CVD risk estimates combined with different recommended risk thresholds for each prediction model, may lead to different definitions of high-risk individuals. For example, the Pooled Cohort Equation stratifies individuals with a > 7.5% 10-year CVD risk as high-risk whereas the recommended threshold for the Framingham risk equation is 10% [8, 9]. Different definitions of high-risk individuals may, in turn, lead to different treatment recommendations. Furthermore, the expected health benefits of treatment may also be different since the impact on quality of life differs per CVD event type and severity. For example, the expected health loss due to a stroke is expected to be higher than the health loss due to a myocardial infarction [10].

Since the implication of different treatment recommendations could be large, the aim of this paper is to assess if the use of different prediction models leads to different treatment recommendations in clinical practice. Therefore, four widely used CVD risk prediction models were investigated regarding their comparability and interpretation, after applying them to a large population cohort. Additionally, we discuss the usefulness of such models based on the comprehensiveness of their composite endpoint and provide a recommendation for the development of new prediction models in order to enhance their usefulness in clinical practice. This paper does not focus specifically on Dutch clinical practice and does not provide guidance on preferred prediction models for the Dutch context.

## Methods

Adult Treatment Panel III (ATP), Framingham Global Risk Score (FRS), Pooled Cohort Equations (PCE), and SCORE-low (SCORE) are four widely used CVD risk prediction models for primary prevention [11–14]. All are derived from general population cohort data. Hence, they include (often similar) predictors that are easy to measure in everyday clinical practice, such as gender, age and systolic blood pressure. The exact definition of the included risk factors in the risk equation can be found in the original publication [11–14]. Furthermore, the probability estimate of each model reflects the absolute risk of the composite endpoint occurring within 10 years. In order to compare these four models, we first identified the exact definition of each composite endpoint from the original publication describing the development of the model [11–14]. We then, standardized the composite endpoints using ICD-10 codes. This was necessary since the published articles often only described the outcomes in words, e.g. “coronary heart disease” or “ischemic heart disease”.

To compare the composite endpoints, we used the MORGEN cohort. The MORGEN cohort is a large Dutch general population cohort which includes men and women aged 20 to 74 years at baseline, recruited from the general population between 1993 and 1997 [15]. Participant information on vital status, cause of death and comorbidity was obtained from Statistics Netherlands and the National Medical Registry (NMR). The follow-up period of the MORGEN cohort was 10 to 15 years with a mean follow-up time of 12 years. To apply the prediction models, information both from baseline and from follow-up was required, leaving 19,484 (72%) individuals with adequate data for the analysis from the original cohort [16, 17]. To further investigate the constitution of the composite endpoint, we determined the observed rates and distributions of the individual components, i.e. included CVD event type according to the associated ICD-10 code(s), for each model *separately*.

As the indication for statin therapy is also LDL-dependent and we aim to illustrate the complexity of CVD risk predictions by comparing results of different prediction models, individuals with an elevated level of LDL and/or diabetes were excluded for further analysis. We focused on individuals in whom preventive intervention was indicated based on predicted CVD risk rather than on elevated LDL levels and/or diabetes. After excluding 231 individuals with diabetes, 1,141 individuals with elevated LDL levels and 25 individuals with both risk factors at baseline, this resulted in a cohort size of 18,137 individuals (mean age = 42.4 years, range 20.1–73.7 years, and 45% men).The MORGEN cohort was also used to compare the predicted CVD risks by estimating every individual’s 10-year CVD risk with each of the four prediction models. Implementation of a prediction model typically follows updating or recalibration of the model in the target setting, as the target cohort may differ from the original development cohort [18]. Therefore, we first recalibrated the four prediction models using the MORGEN cohort to ensure that the models provide accurate risk estimates in this cohort. For the survival data (time-to-event data) considered in this study, recalibrating a prediction model typically involves updating the baseline hazard and centering each predictor around the mean value of all patient characteristics in our empirical cohort, correcting for men and women separately [19, 20]. Furthermore, we incorporated an additional correction factor to ensure that the updated baseline hazards actually reflect the observed probability of survival after 10 years.

Many clinical guidelines advocate the use of prediction models to select individuals with a predicted risk above a certain threshold for preemptive lipid or blood pressure lowering drug treatment. Different recommendations for absolute 10-year risk thresholds were identified for each model: 10% (ATP), 10% (FRS), 7.5% (PCE), and 5% (SCORE) [9, 12, 21]. By doing this, we were able to further explore and compare the varying treatment decisions according to the four models. Finally, we first assigned individuals to treatment based on their FRS risk and the FRS risk threshold and then reassigned individuals according to their ATP, PCE, and SCORE risks, and the corresponding thresholds.

The aim of this paper was to illustrate the complexity of comparing predicted risks. This paper does not focus specifically on Dutch clinical practice and does not provide guidance on preferred prediction models for the Dutch context.

## Results

Although the predictors of the four prediction models are similar, the composite endpoints vary widely and include different CVD event types (Table 1, column 1–6). Myocardial infarction (MI) is included in all four composite endpoints, either alone or in combination with other CVD event types. The endpoint defined for FRS includes the largest range of fatal and non-fatal CVD event types, whereas the endpoint defined for SCORE only includes fatal event types.

**Table 1:** Constitution of the composite endpoints according to ATP, FRS, PCE, and SCORE and incidence of CVD events in MORGEN cohort.

		ATP		FRS		PCE		SCORE	
Individual components	ICD-10 code		#		#		#		#
**Morbidity**									
Myocardial infarction (MI)*[27]	I21,I22	X	183	X	164	X	176		
Other Coronary heart disease (OCHD)	I20,I23,I24,I25			X	348				
Cardiac arrest, sudden death	I46,R96			X	3				
Hemorrhagic stroke (CVAH)	I60,I61,I62			X	39	X	39		
Ischemic stroke (CVAI)	I63,I65			X	56	X	58		
Other stroke (OCVA)	I64,I66			X	29	X	29		
Other Cardiovascular diseases (OCVD)	G45,I67,I69,I70-I74,I50			X	222				
***Total observed events***		***183***		***861***		***302***		***0***	
**Mortality**									
Myocardial infarction (MI)	I21,I22	X	38	X	33	X	38	X	48
Other Coronary heart disease (OCHD)	I20,I23,I24			X	3			X	12
Cardiac arrest, sudden death	I46,R96			X	7			X	8
Hemorrhagic stroke (CVAH)	I60,I61,I62			X	5	X	5	X	12
Ischemic stroke (CVAI)	I63,I65			X	2	X	2	X	4
Other stroke (OCVA)	I64,I66			X	1	X	3	X	2
Other Cardiovascular diseases (OCVD)	G45,I67,I69,I70-I74,I50			X	16			X	19
***Total observed events***		***38***		***67***		***48***		***105***	
**Composite endpoints** ***(morbidity + mortality)***									
Ischemic Heart disease (IHD)	I20-I25								
Coronary heart disease (CHD)	I20-I25,I46,R96								
Cerebrovascular accident (CVA)	I60-I66					*X*			
Cardiovascular disease (CVD)	I20-I26,I46,R96,G45, I60-I67,I69,I70-I74,I50			*X*				*X (only fatal events)*	
***Overall observed events***		***221***		***928***		***350***		***105***	

* The primary endpoint for ATP III is ‘hard CHD’, however model ATP III was based on the previously developed Framingham risk score with total CHD as primary endpoint. For this study, the endpoint defined in the original ATP III paper is followed, i.e. endpoint ‘hard CHD’ is used.

Table 1 (column 4, 6, 8, and 10) shows the incidence of each CVD event type as observed in the MORGEN cohort for the four different prediction models. Due to different composite endpoints, individuals with an earlier CVD event which was not included in the considered endpoint were not censored. Therefore, the observed event rates for a specific CVD event vary per prediction model. Definition of a first and secondary event within individuals thus depends on whether the observed CVD events for individuals are included in the composite endpoint of each prediction model. Due to the different definitions of the composite endpoint of the four prediction models, the total number of observed events for SCORE (n = 105) is almost nine times smaller than for FRS (n = 928). These differences in composite endpoints also affect the absolute number of observed events per prediction model, due to different censoring mechanisms. For example, the absolute number of fatal MIs varies per prediction model because secondary fatal MIs may be censored due to occurrence of another primary event present in the composite endpoint. To illustrate: a fatal MI following a non-fatal stroke event would be accounted for (*not* censored) in the SCORE and ATP model and not accounted for (censored) in the FRS and PCE model. The relative incidence of CVD event types within composite endpoints also varies substantially. For example, of the 105 events observed according to SCORE, 48 (46%) were fatal MIs, whereas the relative incidence of fatal MIs is 17%, 4%, and 11%, for ATP, FRS, and PCE, respectively. This means that the burden, or health loss, associated with the incidence of each composite endpoint varies with a) the included event types, and b) the relative incidence of these event types.

The performance of the recalibrated prediction models was good and quite similar; the c-index was 0.81, 0.78, 0.78, and 0.81 for ATP, FRS, PCE, and SCORE respectively. S2—Table 1 shows an overview of the observed and predicted number of CVD events for each of the four models. Following from this table, it is apparent that the events are well captured by the models and that the number of predicted events closely matches the observed number of events.

**Fig 1:**
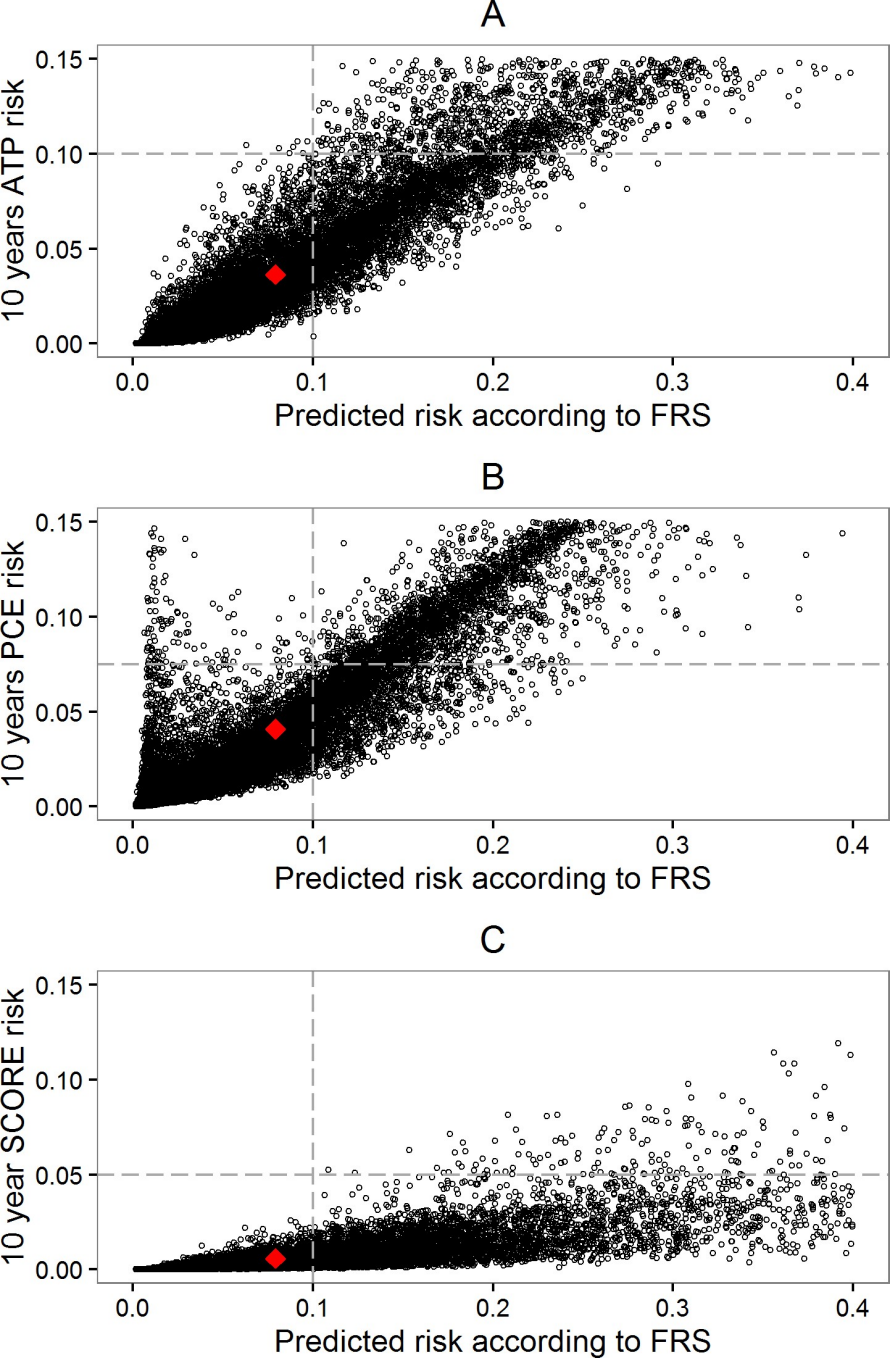
**Predicted (absolute) CVD risk according to FRS and A) ATP, B) PCE, and C) SCORE.** The red marker is the estimate of the mean predicted risk according to FRS and ATP, PCE, or SCORE. The grey lines (raster lines) represent the different risk thresholds and reveal the fraction of individuals eligible for treatment.

Fig 1 shows that the dissimilarities in composite endpoints lead to substantial variation in predicted 10-year CVD risks. Since predicted risks increase with the inclusion of more CVD event types in the composite endpoint, to an extent that depends on their absolute incidence. For example, in our cohort the broad composite endpoint used in the FRS model, covering a large range of CVD event types, yields higher risk predictions than the ATP, PCE, and SCORE models. Similarly, the narrow composite endpoint of SCORE (only fatal events), and its inherent low incidence of included event types yields the lowest risk predictions of all models considered. The average predicted risks in the MORGEN cohort are 1.2% (ATP), 5.1% (FRS), 1.9% (PCE), and 0.6% (SCORE). Hence, the differences in composite endpoints between prediction models, shown in Table 1, result in large variation in predicted CVD risks across prediction models.

Considering that the largest set of CVD event types was included by the FRS composite endpoint, we compared FRS risk estimates with risk estimates from the other three models using more narrow composite endpoints. Fig 1 shows the comparison in CVD risks and reveals an association between these risk estimates. This association indicates that, *in this cohort*, individuals who have the highest risk according to FRS typically also have the highest risk according to ATP, PCE, and SCORE. However, while the *relative* risks are similar the *absolute* risks are clearly different. Furthermore, the vertical spread of points in Fig 1 shows how individuals with a certain FRS risk estimate may have varying risk estimates according to the other models, due to the effect of different risk factor combinations in each model. For example, the group of individuals with an average predicted FRS risk of 10% had an average PCE risk of 3.9%, with a 95% percentile range of 2.2%-5.1% (Fig 1, plot B).

Given the variation in composite endpoints and the subsequent variations in risk predictions from the four models, selecting high risk individuals based on the corresponding recommended risk thresholds results in highly different high risk groups, identified per model. Unfortunately, the fact that each prediction model has its own associated risk threshold further complicates the interpretation and comparison of absolute predicted risks between prediction models. Consequently, treatment decisions may vary with the prediction model that is used. For example, in the MORGEN cohort these thresholds would possibly lead to a seventy-fold difference in prescription of preventive drug treatment in 0.2%, 14.4%, 4.3%, and 1.4% of all individuals, when using ATP, FRS, PCE and SCORE, respectively. To illustrate the implications of these differences, we determined the CVD risks and the consequences on treatment decisions according to the four prediction models for one individual in our cohort. Indeed, using FRS for this individual implies both a greater necessity to consider preventive drug treatment and a larger potential benefit of such treatment, compared to ATP, PCE, and SCORE (see S1 Appendix—Clinical example).

The treatment decisions based on the four risk thresholds are shown in Table 2. We found that the treatment decisions based on the different models vary widely, which is undesirable from a public health point of view. When using FRS, 2618 individuals have an estimated risk exceeding the FRS threshold and would thus be eligible for medical treatment. Of these individuals, only 32 (1.2%), 725 (27.7%), and 56 (2.1%) individuals would be considered eligible for medical treatment using the estimated risk and corresponding threshold when applying ATP, PCE, and SCORE respectively.

**Table 2:** Reclassification table where all individuals are classified and considered for treatment, according to the FRS risk threshold (10%), and reassigned for treatment according to the thresholds according to ATP (10%), PCE (7.5%), and SCORE (5%).

		Framingham risk prediction (percentiles)	
		Below (risk < 10%) N = 15,519	Above (risk ≥ 10%) N = 2618
	Mean predicted risk	3.26%	16.15%
	Observed events	489	439
**Reclassification**			
**ATP**	Mean predicted risk	0.68%	4.42%
	Observed events	209	12
		N (%)	N (%)
	Below (risk < 10%)	15,519 (100)	2586 (98.78)
	Above (risk ≥ 10%)	0 (0)	32 (1.22)
		
**PCE**	Mean predicted risk	1.17%	6.44%
	Observed events	172	178
		N (%)	N (%)
	Below (risk < 7.5%)	15,469 (99.68)	1894 (72.35)
	Above (risk ≥7.5%)	50 (0.32)	724 (27.65)
		
**SCORE**	Mean predicted risk	0.55%	0.76%
	Observed events	87	18
		N (%)	N (%)
	Below (risk < 5%)	15,330 (98.78)	2562 (97.86)
	Above (risk ≥ 5%)	189 (1.22)	56 (2.14)

All individuals are separated into two subgroups “below” and “above” based on the FRS risk threshold, with the following definitions; ***below****—*individuals with a predicted risk < 10% (no treatment), and ***above******—***individuals with a predicted risk ≥ 10% (treatment). For each (FRS-)subgroup (column 3–4), the number of individuals present (N) and their average predicted FRS risk (%) is shown. For each FRS-subgroup, individuals are reassigned into two (sub-)subgroups below or above according to ATP (row 5–8), PCE (row 10–13), and SCORE (row 15–18).The green highlighted cells indicate *concordance* and blue highlighted cells indicate *discordance* on the classification of individuals.

These different decisions may be due to either the different estimated risks or due to the use of different risk thresholds for classifying individuals as high risk and thus eligible for medical treatment. In our cohort, we observed that mostly the same individuals were assigned a *relatively* high risk according to each of the four prediction models (Fig 1). For example, of the individuals with the highest 20% predicted risks according to FRS (n = 3621), 3106 (85.8%), 3131 (86.5%), and 861 (23.8%) of individuals were also classified as relatively high risk (top 20%) according to ATP, PCE, and SCORE, respectively. This relatively high risk group had an average CVD risk of 14.2% according to FRS, and average risks of 3.9%, 5.6%, and 0.7% according to ATP, PCE, and SCORE, respectively. None of the individuals within the top 20% risk group according to FRS had a relatively low risk (bottom 20%) according to the other models. Hence, the expected differences in treatment decisions across prediction models is mainly due to the different corresponding treatment thresholds, and their relation to predicted risks, and not due to the different classification of individuals.

## Discussion

CVD risk prediction is key in providing preventive medication to large groups of individuals at intermediate or high risk of future CVD events, despite absence of specific elevated risk factors. Although PCE is often used, contemporary decision making and CVD management in the US, FRS is also applied, for example to guide pharmacotherapy for LDL-C lowering in women [9].

This paper illustrates the complexities of interpreting and comparing predicted 10-year CVD risks from four widely used CVD risk prediction models. We showed that the models vary substantially regarding their composite endpoints, and therefore also regarding their predicted absolute risks. As a result, absolute predicted 10-year risks from different prediction models cannot be compared directly and treatment decisions depend on the applied prediction model and its associated risk threshold. For example, of the high-risk individuals considered for preventive treatment according to FRS, only 1%, 28%, and 2% were eligible according to ATP, PCE, and SCORE, respectively (Table 2). Hence, the choice for a specific prediction model is very likely to impact treatment decisions in a large group of assessed individuals. Fortunately, the variation in relative predicted CVD risks is limited, implying that these prediction models rank individuals similarly regarding their CVD risk.

### Consequences of difference in composite endpoints on clinical utility

Previous research has indicated that the use of composite endpoints instead of single endpoints *in clinical trials* may have benefits, e.g. improved power or wider coverage of the disease [22]. However, the overall usefulness of composite endpoints in clinical trials is still debated due to the difficulty of interpreting differences in ‘set of outcomes’ [22, 23]. The interpretation of the associated consequences of predicted CVD risks is also directly affected by the different composite endpoints. For example, communicating to a patient that he/she has a 10-year CVD risk of 3% according to SCORE, compared to a 10-year CVD risk of 6% according to FRS, may affect understanding and adherence of patients to any recommended preventive treatment. A 3% SCORE risk could indicate that the patient is part of the group with the 20% highest absolute risk according to SCORE whereas the patient could be part of the group with the 20% lowest predicted absolute risk with a 6% risk according to FRS (Fig 1).

In addition, the expected health loss due to events predicted by SCORE is expected to be higher than the health burden or health loss due to events predicted by FRS due to how all included events in SCORE are fatal, but can fatal or non-fatal in FRS. This issue also affects the evaluation of benefits from preventive interventions. For example, when preventive statin treatment is assumed to reduce the risk of a “composite” endpoint with a certain fraction (relative risk < 1), estimates of the corresponding health benefits will be highly dependent on the (constitution of) the composite endpoint of the prediction model used [24].

Even for a single prediction model, the impact of experiencing a predicted composite event is likely to depend on age, since a) the proportion of fatal events increases with age, and b) the actual health loss due to CVD events decreases with age (i.e. with decreasing life expectancy). Hence, even if the distribution of events included in a composite endpoint is known, the expected health impact of a specific risk estimate, for example a 10-year FRS risk of 8%, and therefore the potential benefits of preventive intervention, may differ between groups of individuals [25].

Given the adequate performance of the CVD prediction models considered, and roughly similar relative risk classification, it is recommended that models are applied that have a broad rather than narrow composite endpoint, i.e. models covering a large range of CVD event types. For example, ATP and SCORE may be less useful in this context than FRS and PCE, as the latter cover more manifestations of the underlying cardiovascular disease process. This results in higher predicted risks, which may then be communicated as the ‘total risk’ of any (type of) CVD event to the patient, to facilitate understanding and improve adherence to preventive medication [26]. However, understanding the “total (high) risk” is only an aspect of adherence and should not replace informed choice and shared decision making.

### Implications for development of new prediction models

Regarding prediction model development and research, it is recommended that any newly developed clinically relevant risk prediction model also use a broad composite endpoint, with each included event type uniquely defined, e.g. using ICD-10 codes. A clear definition of a) the composite endpoint and b) the observed incidence of each event type in the development cohort is critical to enable correct interpretation of the predicted risks. This will allow for more transparent and direct comparison of predicted risks and statistical performance of prediction models as well as more standardized evaluations of the health impact of risk-based preventive interventions.

## Conclusion

Current CVD risk prediction models vary widely in predicted outcomes, which directly impact their usefulness in clinical practice. Furthermore, this renders estimates of the population burden of CVD, and of the impact of risk-based CVD intervention strategies that highly depend on the prediction model used. Physicians, patients and health policy makers may benefit from a broader and more standardized method of defining outcomes and classification thresholds in prediction model studies.

## Supporting information

S1 Appendix (PDF)

S2 Appendix (PDF)
