# Supplementary material for: Interpretation of CVD risk predictions in clinical practice: Mission impossible?
Source: PLoS One. 2019 Jan 9;14(1):e0209314. doi: 10.1371/journal.pone.0209314 (PMC6326414; doi:10.1371/journal.pone.0209314)
Supplement: S1 Appendix — (PDF) [file pone.0209314.s001.pdf]

S1 Appendix

Clinical example

Consider a 57 year-old female, with total cholesterol, low-density-lipoprotein (LDL) and high-density-lipoprotein (HDL) levels of 193 mg/dL, 91 mg/dL, and 54 mg/dL, respectively, a systolic blood pressure (SBP) of 186, no use of blood pressure lowering drugs, who does not smoke and does not have diabetes. This woman has an estimated 10-year CVD risk of 1.9%, 14.1%, 3.2%, and 0.4%, according to ATP, FRS, PCE, and SCORE, respectively. This would indicate a risk above the respective treatment threshold for FRS (10%), but not for ATP (10%), PCE (7.5%) and SCORE (5%).

The use of these different risk prediction models may not only lead to different treatment decisions, but also to different estimates of the expected benefit from preventive treatment. When this woman would receive preventive statin treatment, expected to reduce the overall CVD risk with 30%, this would reduce the risk of non-fatal and fatal MI by 0.6% according to ATP, whereas according to SCORE, the risk of fatal CVD events would decrease by 0.1% [1]. Similarly, the risk of non-fatal and fatal MI and stroke would decrease by 1.0% according to PCE, whereas according to FRS, the risk of the broad range of CVD events included in FRS, both non-fatal and fatal, would be reduced by 4.2%. Apparently, for this woman CVD risk prediction using FRS implies both a greater necessity to consider preventive drug treatment and a larger potential benefit of such treatment, compared to ATP, PCE, and SCORE.

1. Taylor, F., et al., *Statins for the primary prevention of cardiovascular disease.* Cochrane Database Syst Rev, 2013(1): p. CD004816.
