# Supplementary material for: Interpretation of CVD risk predictions in clinical practice: Mission impossible?
Source: PLoS One. 2019 Jan 9;14(1):e0209314. doi: 10.1371/journal.pone.0209314 (PMC6326414; doi:10.1371/journal.pone.0209314)
Supplement: S2 Appendix — (PDF) [file pone.0209314.s002.pdf]

S2 Appendix

S2 Table 1: Observed and predicted number of CVD events

	# Observed CVD events	# Expected CVD events		
		Original model	Recalibrated model	Recalibrated model with correction factor
ATP	221	581	672	221
FRS	928	1147	1456	928
PCE	350	577	751	350
SCORE	105	119	105	105
